# Role of ^18^F-FDG Positron Emission Tomography/Computed Tomography Imaging in Testicular Lymphoma

**DOI:** 10.4274/mirt.galenos.2018.04127

**Published:** 2019-03-19

**Authors:** Kamal Kant Sahu, Ajay Mishra, James O’shea

**Affiliations:** 1 Department of Internal Medicine, Saint Vincent Hospital, 123 Summer Street, Worcester, MA, 01608, United States; 2 Division of Hematology and Medical Oncology, Saint Vincent Cancer and Wellness Center, 1 Eaton Place, 01608, Worcester USA

**Keywords:** Testis, DLBCL, R-CHOP, PET scan

## Dear Editor,

We read with great interest the recent article by Okuyucu et al. (1) regarding the role of ^18^F-FDG PET/CT in the management of testicular lymphoma. Hereby, we would like to share our experience with regards to the diagnostic approach in a case of testicular lymphoma.

^18^F-FDG PET/CT scan has a pivotal role as an initial modality to investigate non-Hodgkin’s lymphoma. PET scan is now even considered the standard of care in follow-up, to assess the response and for tailoring the subsequent therapy. Thanks to the newer diagnostic modalities, oncologists are now diagnosing malignancies in rare sites as well (2,3,4). It is important to note that due to rare location, there are no standard guidelines to follow and, in such situations, oncologists investigate and treat based on their individual experience and available literature. 

Testicular lymphoma is unique with regards to its location, aggressive nature and high rate of relapse to contralateral testis/central nervous system. In the testis, unlike the other sites, fine needle aspiration cytology and biopsy are not considered as the diagnostic tool and orchiectomy has both diagnostic and therapeutic implications PET scan provides essential information about the side of involvement (unilateral vs bilateral), extent and pattern of disease involvement (intense, mild, focal diffuse SUV uptake), risk of relapse (SUV_max_ uptake in brain parenchyma or contralateral testis), need of intrathecal methotrexate, radiation therapy to the contralateral testis etc.

Sidhu et al. (5) recently mentioned the different patterns of ^18^F-FDG uptake (i.e. normal, focal, multifocal, symmetrically diffuse, asymmetrically diffuse) in their institutional study of 12 cases of lymphoma with secondary testicular involvement. Important to note that five out of 12 patients in their study had concurrent CT scans which were reported as normal. This fact again signifies the impeccable role of PET/CT. Recently, Ollila and Olszewski (6) studied the role of radiotherapy in primary testicular lymphoma. PET/CT would again be a good tool to guide radiation oncologists to determine the radiation field.

Radiotherapy, addition of rituximab, prophylactic intrathecal chemotherapy and use of PET/CT scan have certainly improved progression free survival and overall survival in testicular lymphoma. Till today, data regarding testicular lymphoma are mostly derived from small case series and retrospective studies. Involvement of extramedullary sites especially reproductive organs can be extremely challenging due to their masquerading, atypical clinical presentations and impact to fertility  (7,8,9). More studies and randomized clinical trials are required and would be helpful in formulating uniform guidelines for the management of testicular lymphoma.
